# Outdoor air pollution and diminished ovarian reserve among infertile Korean women

**DOI:** 10.1186/s12199-021-00942-4

**Published:** 2021-02-11

**Authors:** Hannah Kim, Seung-Ah Choe, Ok-Jin Kim, Sun-Young Kim, Seulgi Kim, Changmin Im, You Shin Kim, Tae Ki Yoon

**Affiliations:** 1 Department of Obstetrics and Gynecology, CHA Fertility Center Seoul Station, CHA University School of Medicine, Seoul, 04637 Korea; 2 Department of Preventive Medicine, Korea University College of Medicine, Seoul, 02841 South Korea; 3 Department of Epidemiology & Health Informatics, Graduate School of Public Health, Korea University, 73 Goryeodae-ro, Seongbuk-gu, Seoul, 02841 Korea; 4 Department of Cancer Control and Population Health, Graduate School of Cancer Science and Policy, National Cancer Center, Goyang-si, Gyeonggi-do 10408 Korea; 5 Department of Public Health Science, Graduate School of Public Health, Seoul National University, Seoul, 08826 Korea; 6 Department of Geography, Korea University, Seoul, 02841 South Korea

**Keywords:** Air pollution, Ovarian reserve, Anti-Müllerian hormone, Fertility

## Abstract

**Background:**

Mounting evidence implicates an association between ambient air pollution and impaired reproductive potential of human. Our study aimed to assess the association between air pollution and ovarian reserve in young, infertile women.

**Methods:**

Our study included 2276 Korean women who attended a single fertility center in 2016–2018. Women’s exposure to air pollution was assessed using concentrations of particulate matter (PM_10_ and PM_2.5_), nitrogen dioxide (NO_2_), carbon monoxide (CO), sulfur dioxide (SO_2_), and ozone (O_3_) that had been collected at 269 air quality monitoring sites. Exposure estimates were computed for 1, 3, 6, and 12 months prior to the ovarian reserve tests. Anti-Müllerian hormone (AMH) ratio (defined as an observed-to-expected AMH based on age) and low AMH (defined as < 0.5 ng/mL) were employed as indicators of ovarian reserve. We included a clustering effect of 177 districts in generalized estimating equations approach. A secondary analysis was conducted restricting the analyses to Seoul residents to examine the association in highly urbanized setting.

**Results:**

The mean age was 36.6 ± 4.2 years and AMH level was 3.3 ± 3.1 ng/mL in the study population. Average AMH ratio was 0.8 ± 0.7 and low AMH was observed in 10.3% of women (*n*=235). The average concentration of six air pollutants was not different between the normal ovarian reserve and low AMH groups for all averaging periods. In multivariable models, an interquartile range (IQR)-increase in 1 month-average PM_10_ was associated with decrease in AMH ratio among total population (*β*= −0.06, 95% confidence interval: −0.11, 0.00). When we restrict our analysis to those living in Seoul, IQR-increases in 1 and 12 month-average PM_2.5_ were associated with 3% (95% CI: −0.07, 0.00) and 10% (95% CI: −0.18, −0.01) decrease in AMH ratio. The ORs per IQR increase in the six air pollutants were close to null in total population and Seoul residents.

**Conclusions:**

In a cohort of infertile Korean women, there was a suggestive evidence of the negative association between ambient PM concentration and ovarian reserve, highlighting the potential adverse impact of air pollution on women’s fertility.

**Supplementary Information:**

The online version contains supplementary material available at 10.1186/s12199-021-00942-4.

## Background

Ovarian reserve is an important indicator of female reproductive potential. It is largely determined by the number and quality of oocytes in the ovaries [1]. Generating oocytes or proceeding to atresia, the number of follicles in the ovaries declines over time because they are not restored [2]. When women are in their late 30s or early 40s, the decrease in follicle pool starts to accelerate and they reach menopause 10–15 years later [3, 4]. In 10–26% of women, ovarian reserve is more diminished than age-related decrease and it can lead to earlier menopause [5–9]. Causes of this diminished ovarian reserve (DOR) remain unknown in most cases [7].

Several epidemiological and clinical studies have suggested that a woman’s ovarian reserve is influenced by environmental exposures. For example, heavy smoking, long-term exposure to secondhand smoking, and indoor burning of wood or artificial fire logs have been found to be associated with decreased ovarian reserve in some studies [10, 11]; however, these findings were not replicated in other studies [12, 13]. Environmental exposure to endocrine disrupting chemicals such as parabens was reported to be negatively associated with ovarian reserve [14]. Recent reports corroborate that exposure to higher levels of PM is associated low ovarian reserve parameters [15–17]. However, these findings are limited by the small study population. This study assessed the association between air pollution and age-adjusted measures of ovarian reserve in a cohort of women who visited a fertility center in Seoul, Republic of Korea.

## Methods

### Study population

Data of infertile women who visited a single fertility center for fertility evaluation was used. The center is the largest single fertility center of the country located in Seoul, South Korea, and a half of patient population are from outside of the capital area (9 provinces of the country). The result of ovarian reserve test and residential address of those who newly visited between January 2016 and September 2018 were obtained. Because our exposure assessment is based on air quality monitoring data, our analysis was restricted to the subjects living within 6 km from a monitoring station to minimize measurement error [18–20]. Having further excluded women previously diagnosed with chromosomal abnormality, having a history of unilateral or bilateral oophorectomy, and aged < 20 or > 49 years, the final study population included 2276 women.

### Estimation of ambient air pollution exposure

Hourly concentrations of particulate matter less than or equal to 10 or 2.5 μm in diameter (PM_10_ and PM_2.5_, respectively), nitrogen dioxide (NO_2_), carbon monoxide (CO), sulfur dioxide (SO_2_), and ozone (O_3_) measured at the 269 air quality monitoring sites located throughout the country for 2016 and 2018 were used. PM_10_ and PM_2.5_ are inhalable particles containing chemical compounds which can reach deep into the lungs and even into the bloodstream [21]. These data were from the National Institute of Environmental Research (NIER, https://www.nier.go.kr/). The following daily representative concentrations of the six pollutants were determined for each woman: 24-h averages for PM_10_, PM_2.5_, NO_2_, and SO_2_, and maximum of seventeen 8-h moving averages for CO and O_3_. In order to obtain consistent measurements, the daily averages calculated only for days in which > 75% of hourly measurements (18 h) were used at each site. The maximum concentrations of CO and O_3_ were used because the majority of their production is affected by commuter traffic and sunlight, respectively [22]. Using these daily representative concentrations, average concentrations for the four periods of 1, 3, 6, and 12 months before the ovarian reserve test were computed. These four periods of exposure allow us to explore the critical period of exposure, given the immediate and long-term change in ovarian reserve was observed when ovaries are affected by chemical injury [23, 24]. The exposure estimate of each period at the nearest monitoring sites was assigned to the women based on their geocoded home addresses at the time of the test assuming women’s addresses remained the same within a year.

### Outcome variable: measurement of ovarian reserve

Anti-Müllerian hormone (AMH) is a widely used indicator of ovarian reserve in women of reproductive age [25]. Those with serum AMH as low as 0.5–1.1 ng/mL are likely to respond poorly to ovarian stimulation and thereby show low pregnancy rates in assisted reproductive technology treatments [26]. In addition, DOR manifested as low AMH has predictive values for the risk of cardiovascular disease [27, 28]. The ovarian reserve of each woman was determined by measuring levels of AMH. Serum obtained on menstrual day 2 or 3 was separated from peripheral blood by centrifugation and stored at − 80 °C until analysis. AMH was measured using the Elecsys® AMH assay (Roche Diagnostics), which is a sandwich assay based on electrochemiluminescence technology. The total duration of the assay is 18 min; the sample volume is 50 μL. The assay is calibrated against the Beckman Coulter AMH Gen II ELISA assay with a measuring range of 0.01–23 ng/mL. Considering the age-dependent change in AMH, the AMH ratio, defined as observed AMH divided by age-specific AMH level, was used as an age-adjusted measure of ovarian reserve [29]. The age-specific reference level of AMH was calculated using a previously described quadratic model (logAMH = −1.442 + 0.225 × age − 0.004 × age^2^) [30, 31]. Given the lower limit of AMH in the minimal criteria for DOR is 0.5 ng/mL [32], further examination was conducted for the risk of AMH < 0.5 ng/mL (“Low AMH”) [33, 34].

### Covariates

Information of women’s age, bodyweight, height, previous smoking history, working status, and residential address was retrieved from medical records. Bodyweight and height were measured at the time of initial visit. Body mass index (BMI) was calculated by dividing person’s weight in kilograms with their height in meters squared and categorized it into one of three groups (low, normal, overweight, and obese) based on the recommended BMI cut-off points for determining overweight and obesity in Asian populations [35]. History of smoking and working status were recorded as binary variables (yes or no).

### Statistical analysis

Descriptive statistics were calculated for the total population over the country. Given women living in capital area may be less deprived socioeconomically and exposed to higher air pollution compared to those living outside, a secondary analysis was conducted restricting to those living in Seoul. The characteristics between Seoul residents and the others were compared. Pairwise correlation structure between air pollutants for different averaging periods was examined with Spearman correlation test. We conducted generalized linear regression analyses for the AMH ratio and logistic regression analyses for low AMH controlling for woman’s age. Effect estimates are presented as regression coefficients for AMH ratio and odds ratios (ORs) for presence of low AMH with their 95% confidence intervals (95% CIs) per an interquartile range (IQR) increment in each pollutant concentration. The IQRs were 8.0 μg/m^3^ for PM_10_, 2.4 μg/m^3^ for PM_2.5_, 8.8 ppb for NO_2_, 1.2 ppb for SO_2_, 870.0 ppb for CO, and 11.0 ppm for O_3_. Risk estimates were adjusted for age (excluded in models for AMH ratio which already adjusted for age), BMI, season at the time of testing, previous smoking history, and district of residence in all models. Age in years (< 35, 35–40, or ≥40 years), BMI, working status, history of smoking (yes or no), and season (March to May, June to August, September to November, or December to February) were recorded as categorical variables. The clustering effect of 177 administrative districts was also included in the model using the *cluster* function of the R package “survival.” The AIC and residual deviance of the generalized linear model (which includes all covariates) for AMH ratio were 4498.6 and 865.23. The AIC and residual deviance were 4448.8 and 856.09 when 1-month average PM_10_ is added to the model. We fitted generalized additive models with non-parametric smoothing splines to further assess a non-linear exposure–response relationship between air pollutant concentration and AMH ratio. The model was fitted with *gam* function of package “gam.” We conducted all the analyses in R (R Version 3.2.1).

## Results

The 2276 women who constituted our study population predominantly were working at the time of the ovarian reserve test (62.9%), had normal weight (62.8%), and reported no history of smoking (97.8%) (Table 1). The mean age was 36.6 (standard deviation= 4.2) years, and AMH level was 3.3 (3.1) ng/mL in total population. Average AMH ratio was 0.8 (0.7) and AMH < 0.5 ng/mL was observed in 10.3% (*n*=235) of total population and 8.6% (*n*=81) of Seoul residents showing higher ovarian reserve in Seoul residents than non-Seoul residents. For a given air pollutant, the 1- and 3-month averages were highly correlated (Spearman’s correlation coefficient= 0.74–0.87) (Supplementary Figure 1). PM_10_, PM_2.5_, NO_2_, and CO concentrations showed positive correlations, whereas O_3_ was negatively correlated with all the other pollutants. When comparing between the normal ovarian reserve and low AMH groups, BMI, proportion of smoking, working status, and average concentration of six air pollutants for all averaging periods were not different (Supplementary Table 1). Women in the low AMH group were older and more likely to live in Seoul.

**Table 1:** Demographic and clinical characteristics and anti-Müllerian hormone (AMH) of the study population, total population (*n*=2,276), Seoul (*n*=1,122), and non-Seoul (*n*=1,154) residents

	Total population (*n*=2276)	Seoul residents (*n*=1122)	Non-Seoul residents (*n*=1154)	*P* for difference^b^
Age (year)	36.6 ± 4.2	36.2 ± 4.1	37.0 ± 4.3	< 0.001
BMI (kg/m^2^)	21.7 ± 3.1	21.4 ± 3.0	22.1 ± 3.3	0.826
< 18.5	243 (10.7%)	129 (11.5%)	114 (9.9%)	< 0.001
18.5–23	1431 (62.8%)	746 (66.5%)	685 (59.4%)	
23–25	300 (13.2%)	128 (11.4%)	172 (14.9%)	
≥ 25	302 (13.3%)	119 (10.6%)	183 (15.8%)	
Timing of blood test				
Mar–May	750 (33.0%)	358 (31.9%)	392 (34.0%)	0.622
June–Aug	489 (21.5%)	252 (22.5%)	237 (20.5%)	
Sept–Nov	337 (14.8%)	171 (15.2%)	166 (14.4%)	
Dec–Feb	700 (30.8%)	341 (30.4%)	359 (31.1%)	
Smoking history	51 (2.2%)	28 (2.5%)	23 (2.0%)	0.584
Currently working	1431 (62.9%)	783 (69.8%)	648 (56.2%)	< 0.001
AMH (ng/mL)	3.3 ± 3.1	3.5 ± 3.2	3.1 ± 3.0	0.001
AMH ratio^a^	0.8 ± 0.7	0.8 ± 0.7	0.8 ± 0.7	0.055
Low AMH (< 0.5 ng/mL)	235 (10.3%)	81 (8.6%)	154 (13.3%)	0.025

*BMI* body mass index, *AMH* anti-Müllerian hormone

^a^Ratio of observed AMH to age-specific AMH. Age-specific AMH was calculated from a quadratic model (logAMH = −1.442 + 0.225 × age − 0.004 × age^2^) [30]

^b^*P* values for difference between Seoul residents and non-Seoul residents. Wilcoxon rank sum test and chi-square test were used for comparison

Due to missing information, sum of columns may not equal to the number of total population

In multivariable models, an interquartile range (IQR) increase in 1-month average PM_10_ was associated with a decrease (beta coefficient = −0.06, 95% confidence interval: −0.11, 0.00, Table 2) in AMH ratio. Overall associations between air pollutants and AMH ratio were toward negative, except ozone, although the estimates did not reach statistical significance. The effect size was similar across the different averaging periods in total population.

**Table 2:** Change in AMH ratio^a^ per interquartile range (IQR)-increase in six air pollutant concentrations among total population (*n*=2276) and Seoul residents (*n*=1122)

Air pollutants/period	Total population (*n*=2276)		Seoul (*n*=1122)	
	Coefficient (95% CI)^b^	*P*	Coefficient (95% CI)^b^	*P*
PM_10_				
1 month-average	**−0.06 (−0.11, 0.00)**	**0.035**	**−0.05 (−0.08, −0.01)**	**0.014**
3 month-average	**−**0.03 (**−**0.09, 0.04)	0.401	**−**0.03 (**−**0.08, 0.02)	0.195
6 month-average	**−**0.02 (**−**0.09, 0.04)	0.483	**−**0.02 (**−**0.08, 0.04)	0.438
12 month-average	**−**0.05 (**−**0.10, 0.01)	0.106	**−**0.07 (**−**0.14, 0.00)	0.056
PM_2.5_				
1 month-average	**−**0.03 (**−**0.09, 0.03)	0.353	**−0.03 (−0.07, 0.00)**	**0.035**
3 month-average	**−**0.01 (**−**0.09, 0.07)	0.777	**−**0.02 (**−**0.07, 0.02)	0.348
6 month-average	**−**0.01 (**−**0.09, 0.06)	0.707	**−**0.03 (**−**0.09, 0.03)	0.364
12 month-average	**−**0.03 (**−**0.08, 0.02)	0.237	**−0.10 (−0.18, −0.01)**	**0.024**
NO_2_				
1 month-average	**−**0.05 (**−**0.12, 0.01)	0.093	**−**0.03 (**−**0.08, 0.02)	0.234
3 month-average	**−**0.05 (**−**0.11, 0.02)	0.143	**−**0.02 (**−**0.07, 0.03)	0.348
6 month-average	**−**0.04 (**−**0.10, 0.01)	0.142	**−**0.02 (**−**0.07, 0.04)	0.532
12 month-average	**−**0.04 (**−**0.09, 0.00)	0.061	**−**0.02 (**−**0.07, 0.03)	0.387
SO_2_				
1 month-average	**−**0.01 (**−**0.06, 0.05)	0.838	**−**0.02(**−**0.07, 0.03)	0.399
3 month-average	0.00 (**−**0.05, 0.05)	0.999	**−**0.01(**−**0.06, 0.05)	0.805
6 month-average	**−**0.03 (**−**0.08, 0.02)	0.258	**−**0.02 (**−**0.09, 0.04)	0.525
12 month-average	**−**0.02 (**−**0.08, 0.05)	0.621	0.00 (**−**0.09, 0.08)	0.934
CO				
1 month-average	**−**0.03 (**−**0.09, 0.03)	0.327	**−**0.03 (**−**0.06, 0.00)	0.084
3 month-average	**−**0.02 (**−**0.07, 0.04)	0.607	**−**0.02 (**−**0.06, 0.02)	0.278
6 month-average	**−**0.03 (**−**0.09, 0.03)	0.266	**−**0.02 (**−**0.06, 0.03)	0.460
12 month-average	**−**0.03 (**−**0.07, 0.01)	0.158	**−**0.03 (**−**0.07, 0.02)	0.250
O_3_				
1 month-average	0.03 (**−**0.06, 0.12)	0.480	0.02 (**−**0.02, 0.06)	0.250
3 month-average	**−**0.02 (**−**0.10, 0.06)	0.638	0.00 (**−**0.04, 0.05)	0.827
6 month-average	0.02 (**−**0.05, 0.09)	0.608	0.00 (**−**0.05, 0.04)	0.886
12 month-average	0.02 (**−**0.02, 0.07)	0.329	0.00 (**−**0.05, 0.05)	0.987

*PM*_*10*_ particulate matter, *PM*_*2.5*_ fine particulate matter, *NO*_*2*_ nitrogen dioxide, *CO* carbon monoxide, *SO*_*2*_ sulfur dioxide, *O*_*3*_ ozone, *CI* confidence interval

^a^Ratio of observed AMH to age-specific AMH. Age-specific AMH was calculated from a quadratic model (logAMH = −1.442 + 0.225 × age − 0.004 × age^2^) from prior study [30]

^b^Adjusted for body mass index (BMI), working status, previous smoking history, season at the time of testing, and district of residence. Estimates in bold have *P* values < 0.05

When restricted to those living in Seoul, the negative association between 1 month-average PM_10_ and AMH ratio was consistent. Additionally, IQR increases in 1- and 12-month average PM_2.5_ were associated with 3% (95% CI: − 0.07, 0.00) and 10% (95% CI: −0.18, −0.01) lower AMH ratio, respectively. The associations with NO_2_, SO_2_, CO, and O_3_ concentrations for four averaging periods were close to null.

**Fig. 1:**
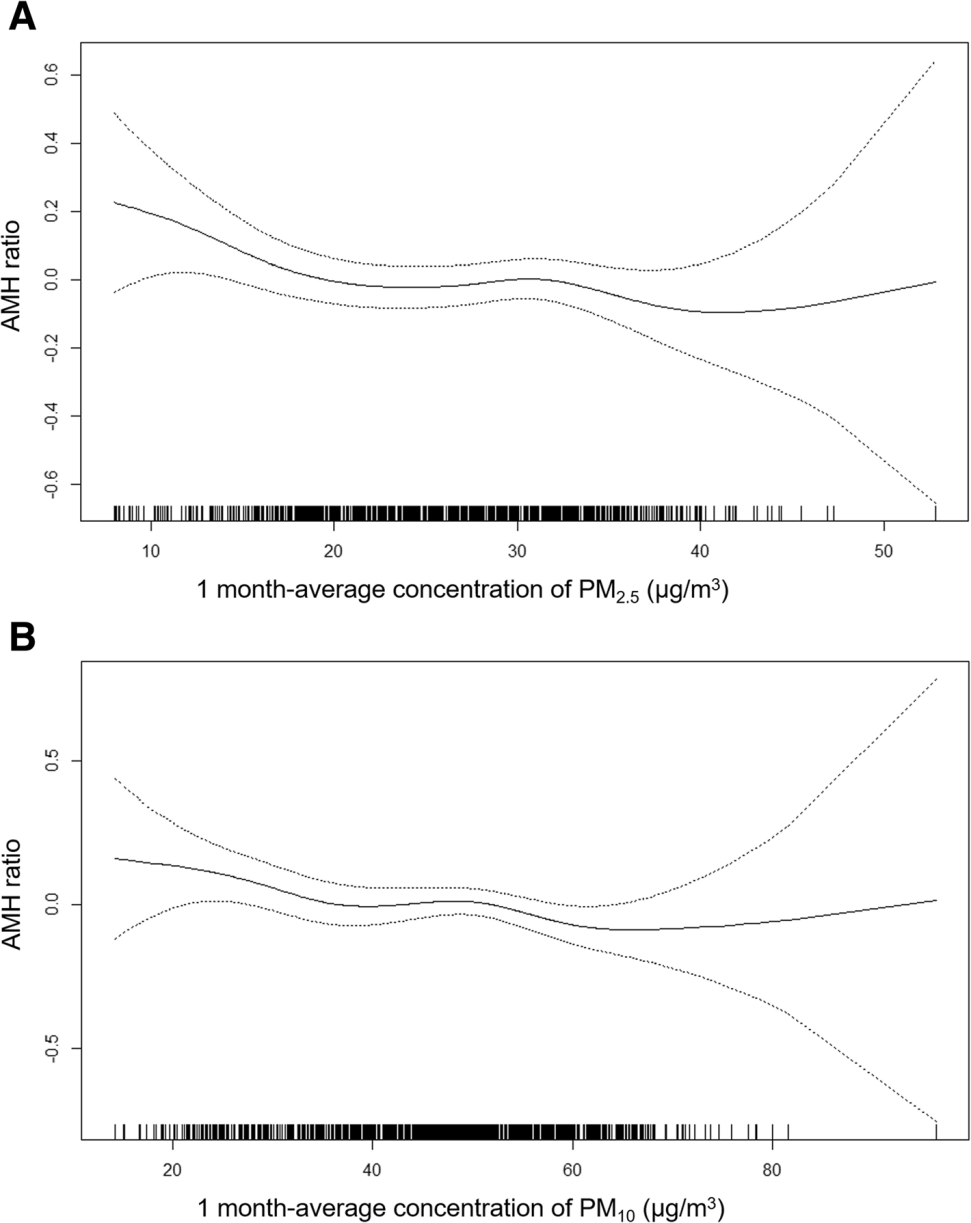
Association between ovarian reserve and air pollutant concentrations. **a** A 1-month average PM_10_ and the AMH ratio in total population and **b** a 1-month average PM_2.5_ and the AMH ratio in Seoul residents. Generalized additive models with non-parametric smoothing splines were used. Interrupted lines indicate 95% confidence intervals of estimates.

For the risk of low AMH, the ORs per IQR increase in the six air pollutants were close to null in total population and Seoul residents (Supplementary table 2). In Seoul residents, a higher 6 month-average CO was associated with lower odds of low AMH with marginal significance (0.80, 95% CI: 0.64, 1.00, *P* = 0.051). For the positive associations of 1-month average PM_10_ (total population) and PM_2.5_ with the AMH ratio (Seoul residents), there was no evidence of important deviations from linearity (Fig. 1).

## Discussion

Our study shows an evidence indicating ambient PM_10_ concentration within a 1-month period is negatively associated with ovarian reserve in women with infertility; also this negative association was additionally observed for PM_2.5_ within 1 month and 12 months in Seoul residents. An association with low AMH levels defined as < 0.5 ng/mL was not evident in our study population. Using a large hospital-based data, we report an association between PM exposure with different averaging periods spanning 1 year and age-adjusted AMH. Although the clinical implication of this short-term impact of air pollution is unclear, this finding may provide additional evidence of potential harm of air pollution among women who are about to undertake fertility treatment.

The negative association between air pollutants and ovarian reserve was present only for PM. It was more evident when the analysis is restricted to Seoul, where the contribution of traffic and secondary aerosol sources is significant in PM generation [36]. Prior studies reported the health effect of air pollution can be greater in the highly polluted cities [37, 38] and traffic-related PM may elicit stronger effect than other source-related one [39]. In accordance with these findings, increased distance from the nearest major road was proportionately associated with higher serum levels of AMH [15]. The relatively high traffic-related PM in Seoul may explain the more consistent association with PM compared to total population over the country.

Although the sensitive windows are unclear, it has been suggested environmental exposure within several weeks or months can be critical in AMH-producing follicular pools. A recent study discovered average concentration of PM_2.5_ within the prior 3 months is associated with decreased antral follicle count [17]. A study of young women exposed to systemic chemotherapy agents showed the reduction of AMH occurs as early as in 15 days following the exposure [23]. Notably, the 12-month average PM_2.5_ also showed negative association with AMH ratio in Seoul residents. Our divergent finding for 1-month and 12-month PM exposure may reflect different mechanisms involved in the association with ovarian reserve between short- and long-term PM exposures.

The causal relationship that links air pollution to ovarian reserve has yet to be elucidated. Prior reports have suggested that folliculogenesis can be impaired by the increased oxidative stress and cellular apoptosis induced by ambient air containing a range of pollutants [40]. In animal studies, exposure to traffic-related air pollution was found to be correlated with a reduction in the number of antral follicles [41–43]. In studies of the adverse effects of smoking on reproduction, lipid peroxidation product—which is associated with accelerated follicle loss in women—was found to be higher in passive smokers than in non-smokers [40, 44]. Even though the causative association of air pollution with low AMH is not yet confirmed, there are some clues as to the nature of its deleterious properties. Unlike other air pollutants, for example, ambient PM contains a number of soluble metals and organic components carried on the particle surface, which play an important role in mediating its toxic effects [45]. This pollutant-specific association would need to be replicated in the future studies.

Our study had some limitations, which ought to be taken into consideration for future studies. First, given the study population is young and infertile women, our finding of an association between air pollution and age-adjusted AMH may not be generalizable to general population. Prior studies on air pollution showed the health-impact of air pollution is generally stronger in more vulnerable population [46]. We believe the infertile patients will more clearly show the association between ovarian reserve and air pollution than general population. Second, our estimations of exposure to ambient air pollution were based on measurements from the nearest monitoring stations, and this may not constitute the actual levels of exposure. More refined measurement would minimize such a potential measurement bias. Third, we did not take account for other ovarian reserve indicators including antral follicle count, follicle-stimulating hormone, and number of oocytes retrieved in in vitro fertilization cycles. Because of high missing rate and low precision for these indicators, we used AMH levels as a sole indicator for ovarian reserve in this study. Studies with other ovarian reserve indicators will add evidence for the association between air pollution and ovarian reserve. Lastly, there is a possibility of false discovery with multiple testing. Given this is one of the limited studies exploring the association between AMH and air pollution, we present all the estimates and their significance using nominal *P* value (=0.05) differing air pollutants and exposure periods to facilitate further researches.

## Conclusions

Using data from a young, infertile women, our study demonstrated that short-term exposures to higher PM levels were associated with lower age-adjusted level of AMH in infertile patients, highlighting the potential adverse impact of air pollution on human fertility and providing further evidence for the same. Further studies involving general population in different geographic and demographic settings are warranted to confirm the findings of this study.

## Supplementary Information

**Additional file 1: Figure S1.** Correlation between the six air pollutants in 206 monitoring sites, 2016-2018. **Table S1.** Clinical characteristics and average concentration of six air pollutants for four exposure periods, normal ovarian reserve versus low AMH groups. **Table S2.** Odds ratios (95% confidence intervals)^a^ of low AMH (< 0.5 ng/mL) per IQR-increase in six air pollutant concentrations in total population (*n*=2,276) and Seoul residents (*n*=1,122).

## Data Availability

The data are not publicly available due to the nature of information that could compromise the privacy of research participants.

## Abbreviations

PM: Particulate matter (PM_10_ and PM_2.5_)
NO_2_: Nitrogen dioxide
CO: Carbon monoxide
SO_2_: Sulfur dioxide
O_3_: Ozone
AMH: Anti-Müllerian hormone
DOR: Diminished ovarian reserve
