# Supplementary material for: Outdoor air pollution and diminished ovarian reserve among infertile Korean women
Source: Environ Health Prev Med. 2021 Feb 11;26:20. doi: 10.1186/s12199-021-00942-4 (PMC7879617; doi:10.1186/s12199-021-00942-4)
Supplement: Supplementary file 1 — Additional file 1: Figure S1. Correlation between the six air pollutants in 206 monitoring sites, 2016-2018. Table S1. Clinical characteristics and average concentration of six air pollutants for four exposure periods, normal ovarian reserve versus low AMH groups. Table S2. Odds ratios (95% confidence intervals)a of low AMH (< 0.5 ng/mL) per IQR-increase in six air pollutant concentrations in total population (n=2,276) and Seoul residents (n=1,122). [file 12199_2021_942_MOESM1_ESM.docx]

Supplementary Figure 1. Correlation between the six air pollutants in 206 monitoring sites, 2016-2018


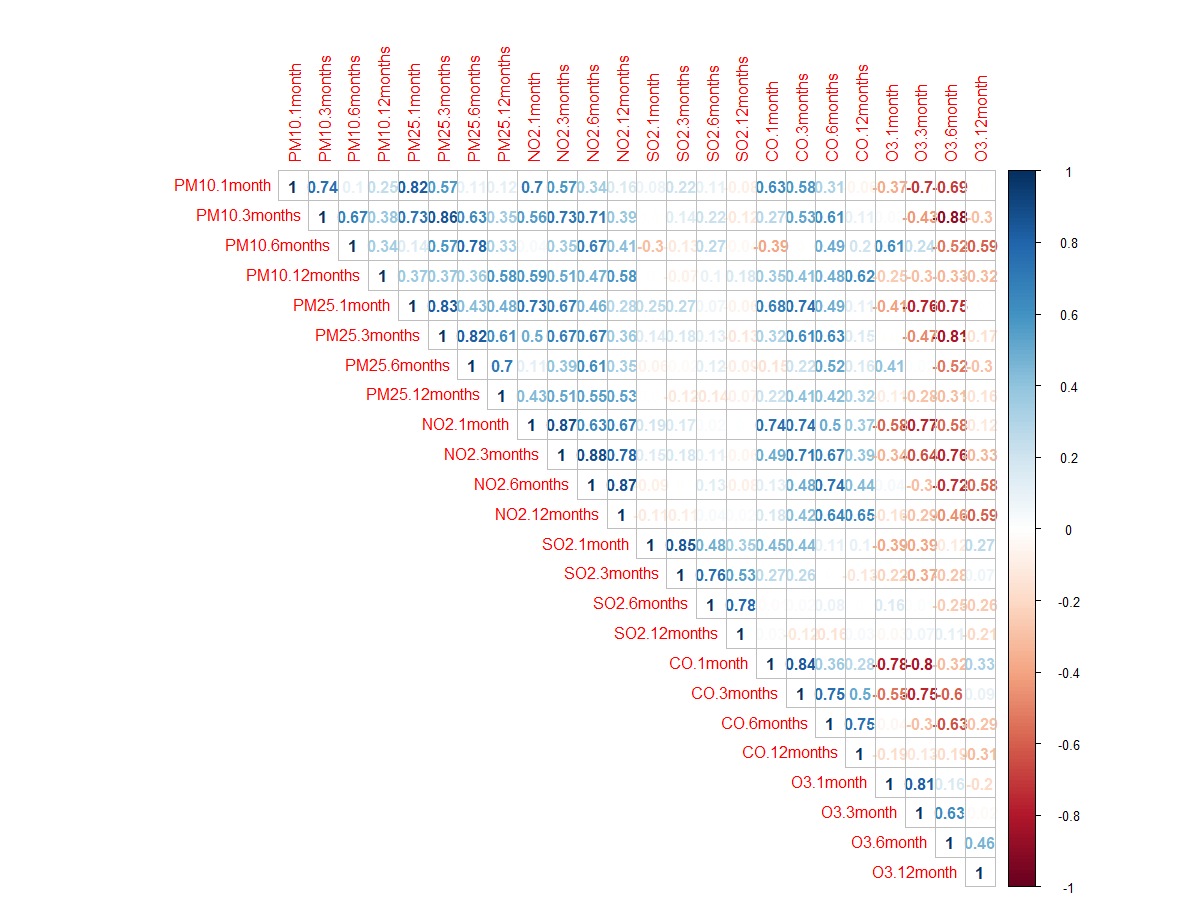


Supplementary Table 1. Clinical characteristics and average concentration of six air pollutants for four exposure periods, normal ovarian reserve versus low AMH groups

|  | Normal ovarian reserve | Low AMH (<0.5ng/mL) | P |
| --- | --- | --- | --- |
|  | % (N) / Mean ± sd | % (N) / Mean ± sd |  |
| Age (year) | 36.2 ± 4.0 | 40.2 ± 4.3 | <0.001 |
| BMI (kg/m^2^) | 21.7 ± 3.2 | 21.8 ± 3.1 | 0.630 |
| Smoking history | 2.3% (46/2,041) | 2.1% (5/235) | 1.000 |
| Currently working | 63.4% (1,294/2,041) | 58.3% (137/235) | 0.126 |
| Living in Seoul | 51.0% (1,040/2,041) | 43.0% (101/235) | 0.025 |
| *Air pollutants* |  |  |  |
| PM_10_ |  |  |  |
| 1 month-average | 46.6 ± 12.4 | 46.2 ± 12.8 | 0.704 |
| 3 month-average | 46.4 ± 10.1 | 45.8 ± 10.4 | 0.375 |
| 6 month-average | 45.1 ± 8.0 | 44.5 ± 8.3 | 0.308 |
| 12 month-average | 46.5 ± 5.9 | 46.7 ± 6.1 | 0.750 |
| PM_2.5_ |  |  |  |
| 1 month-average | 26.2 ± 8.0 | 26.3 ± 8.0 | 0.911 |
| 3 month-average | 26.3 ± 6.4 | 26.4 ± 6.5 | 0.861 |
| 6 month-average | 25.8 ± 5.0 | 25.7 ± 5.3 | 0.880 |
| 12 month-average | 25.7 ± 3.5 | 25.8 ± 3.6 | 0.832 |
| NO_2_ |  |  |  |
| 1 month-average | 29.1 ± 10.3 | 28.5 ± 9.9 | 0.362 |
| 3 month-average | 29.4 ± 9.7 | 28.6 ± 9.4 | 0.206 |
| 6 month-average | 29.2 ± 9.2 | 28.1 ± 9.0 | 0.064 |
| 12 month-average | 29.2 ± 9.2 | 28.1 ± 9.0 | 0.072 |
| SO_2_ |  |  |  |
| 1 month-average | 4.3 ± 1.3 | 4.2 ± 1.2 | 0.211 |
| 3 month-average | 4.3 ± 1.2 | 4.2 ± 1.1 | 0.144 |
| 6 month-average | 4.3 ± 1.1 | 4.2 ± 1.0 | 0.279 |
| 12 month-average | 4.4 ± 1.0 | 4.4 ± 1.0 | 0.701 |
| CO |  |  |  |
| 1 month-average | 62.7 ± 17.9 | 63.5 ± 18.6 | 0.550 |
| 3 month-average | 63.6 ± 16.0 | 64.0 ± 16.6 | 0.763 |
| 6 month-average | 63.0 ± 13.3 | 62.4 ± 13.6 | 0.525 |
| 12 month-average | 62.1 ± 11.0 | 62.1 ± 11.3 | 0.988 |
| O_3_ |  |  |  |
| 1 month-average | 34.6 ± 15.0 | 33.9 ± 14.3 | 0.501 |
| 3 month-average | 33.5 ± 13.0 | 33.4 ± 12.2 | 0.895 |
| 6 month-average | 33.6 ± 10.0 | 34.3 ± 9.6 | 0.352 |
| 12 month-average | 36.7 ± 7.5 | 37.5 ± 7.0 | 0.099 |

PM_10_, particulate matter; PM_2.5,_ fine particulate matter; NO_2_, nitrogen dioxide; CO, carbon monoxide; SO_2_, sulfur dioxide; O_3_, ozone. P values are from Student t tests.

Supplementary Table 2. Odds ratios (95% confidence intervals)^a^ of low AMH (<0.5ng/mL) per IQR-increase in six air pollutant concentrations in total population (n=2,276) and Seoul residents (n=1,122)

| Air pollutants for four exposure periods | Total population (n=2,276) | |  | Seoul residents (n=1,122) | |
| --- | --- | --- | --- | --- | --- |
|  | Adjusted OR (95% CI) | P value |  | Adjusted OR (95% CI) | P |
| PM_10_ |  |  |  |  |  |
| 1 month-average | 0.89 (0.65, 1.21) | 0.444 |  | 0.95 (0.77, 1.17) | 0.901 |
| 3 month-average | 0.77 (0.54, 1.11) | 0.163 |  | 0.77 (0.59, 1.01) | 0.144 |
| 6 month-average | 0.97 (0.67, 1.39) | 0.851 |  | 0.76 (0.53, 1.07) | 0.174 |
| 12 month-average | 1.16 (0.85, 1.58) | 0.335 |  | 1.01 (0.68, 1.52) | 0.882 |
| PM_2.5_ |  |  |  |  |  |
| 1 month-average | 0.95 (0.68, 1.33) | 0.870 |  | 0.99 (0.88, 1.11) | 0.882 |
| 3 month-average | 1.04 (0.69, 1.58) | 0.653 |  | 0.92 (0.79, 1.08) | 0.591 |
| 6 month-average | 1.31 (0.87, 2.00) | 0.157 |  | 0.99 (0.80, 1.22) | 0.844 |
| 12 month-average | 1.15 (0.89, 1.48) | 0.279 |  | 1.14 (0.87, 1.49) | 0.497 |
| NO_2_ |  |  |  |  |  |
| 1 month-average | 0.88 (0.61, 1.25) | 0.639 |  | 0.90 (0.69, 1.19) | 0.442 |
| 3 month-average | 0.79 (0.56, 1.12) | 0.261 |  | 0.83 (0.62, 1.12) | 0.192 |
| 6 month-average | 0.80 (0.59, 1.10) | 0.232 |  | 0.80 (0.58, 1.09) | 0.135 |
| 12 month-average | 0.93 (0.72, 1.20) | 0.648 |  | 0.88 (0.66, 1.18) | 0.280 |
| SO_2_ |  |  |  |  |  |
| 1 month-average | 0.86 (0.64, 1.15) | 0.300 |  | 0.94 (0.71, 1.26) | 0.872 |
| 3 month-average | 0.82 (0.61, 1.12) | 0.212 |  | 0.93 (0.67, 1.28) | 0.927 |
| 6 month-average | 1.09 (0.80, 1.47) | 0.593 |  | 1.14 (0.80, 1.63) | 0.246 |
| 12 month-average | 1.32 (0.93, 1.88) | 0.117 |  | 1.38 (0.89, 2.14) | 0.150 |
| CO |  |  |  |  |  |
| 1 month-average | 0.92 (0.66, 1.27) | 0.603 |  | 0.99 (0.84, 1.16) | 0.913 |
| 3 month-average | 0.82 (0.59, 1.14) | 0.233 |  | 0.88 (0.73, 1.06) | 0.163 |
| 6 month-average | 0.78 (0.56, 1.08) | 0.134 |  | 0.80 (0.64, 1.00) | 0.051 |
| 12 month-average | 0.99 (0.78, 1.24) | 0.903 |  | 0.93 (0.73, 1.18) | 0.547 |
| O_3_ |  |  |  |  |  |
| 1 month-average | 0.90 (0.54, 1.51) | 0.690 |  | 1.09 (0.81, 1.47) | 0.570 |
| 3 month-average | 1.16 (0.73, 1.85) | 0.536 |  | 1.16 (0.84, 1.61) | 0.364 |
| 6 month-average | 1.13 (0.75, 1.70) | 0.567 |  | 1.32 (0.90, 1.93) | 0.154 |
| 12 month-average | 0.91 (0.71, 1.18) | 0.479 |  | 1.15 (0.75, 1.78) | 0.518 |

^a^All estimates were adjusted for age, body mass index (BMI), working status, previous smoking history, season at the time of testing, and districts of residence (177 in total). ORs in bold have P values <0.05.
